# Quantification of overnight urinary gonadotropin excretion predicts imminent puberty in girls: a semi-longitudinal study

**DOI:** 10.1007/s42000-023-00499-7

**Published:** 2023-11-07

**Authors:** And Demir, Atilla Büyükgebiz, Adem Aydin, Matti Hero

**Affiliations:** 1 Pediatric Research Center, New Childrenʼs Hospital, University of Helsinki and Helsinki University Hospital, Biomedicum 2 C, 6th Floor, Tukholmankatu 8 A, FIN-00290 Helsinki, Finland; 2 Department of Pediatrics, Division of Pediatric Endocrinology, Demiroğlu Bilim University, İstanbul, Türkiye; 3 Department of Pediatrics, Dokuz Eylül University Faculty of Medicine, İzmir, Türkiye

**Keywords:** Luteinizing hormone, Follicle-stimulating hormone, Urinary gonadotropin, Onset of puberty, Last-night-voided, First-morning-voided

## Abstract

**Purpose:**

We explored the alternative of using overnight fold change in gonadotropin levels by comparing the last-night-voided (LNV) and first-morning-voided (FMV) urine concentrations of luteinizing hormone (LH) and follicle-stimulating hormone (FSH) as a conceptual analogy to the invasive gonadotropin-releasing hormone (GnRH) stimulation test setting.

**Methods:**

We investigated the nocturnal changes in the immunoreactivity levels of urinary gonadotropins between early and late prepubertal stages as well as between early and late pubertal stages in FMV and LNV urine samples from 30 girls, of whom those who were prepubertal were further investigated through follow-up visits within the 1-year period from the start of the study.

**Results:**

ROC analysis revealed that the FMV total U-LH and FMV U-FSH concentrations at or above 0.3 IU/L and 2.5 IU/L, respectively, were excellent predictors of forthcoming onset of puberty within 1 year (100% sensitivity, 100% specificity, AUC: 1.00, and *n* = 10, for both). FMV total U-LH concentration at or above 0.8 IU/L represented the cut-off for clinical signs of puberty. FMV/LNV total U-LH and FMV/LNV U-FSH ratios at or below 4.11 and 1.38, respectively, were also good predictors of the onset of clinical puberty within 1 year. An overnight increase (FMV/LNV ratio) in total U-LH concentrations and in the U-LH/U-FSH ratio at or below 1.2-fold in pubertal girls was associated with the postmenarcheal pubertal stage.

**Conclusion:**

FMV total U-LH and U-FSH above 0.3 IU/L and 2.5 IU/L, respectively, can be used as cut-off values to predict the manifestation of the clinical signs of puberty within 1 year. FMV total U-LH concentrations 0.3–0.8 IU/L and 0.6 IU/L may represent the range and the threshold, respectively, that reflect the loosening of the central brake on the GnRH pulse generator. An overnight increase of 20% or less in total U-LH concentrations and in the U-LH/U-FSH ratio in an early pubertal girl may serve as an indicator of imminent menarche, a presumed timing of which can be unraveled by future longitudinal studies.

## Introduction

The hypothalamic-pituitary-gonadal (HPG) axis is active during three developmental periods. The axis develops during fetal life is reactivated in the postnatal period during “mini-puberty” and then remains dormant until about 8–9 years of age, with very low concentrations of luteinizing hormone (LH), which can be detected in urine by ultrasensitive LH-specific immunoassays thanks to their ability to detect degraded fractions of LH (the beta subunit and its core fragment) in addition to the intact LH [1–5]. At puberty, a coordinated set of signals allows the reactivation of the axis after this mid-childhood period of dormancy. It is still largely unclear which primary mechanisms suppress the axis after mini-puberty and allow the release of makorin ring finger protein 3 inhibition which acts as a “puberty brake” [6] on neuronal gonadotropin-releasing hormone (GnRH) release until the end of mid-childhood.

LH is secreted in a pulsatile manner well before the onset of puberty, and the gonadotropin secretory pattern characteristic of early puberty results from the amplification of a pre-existing circadian pattern of gonadotropin pulsatility [7, 8]. Indeed, our previous study showed that the nocturnal increase in LH secretion at the onset of puberty can be detected in first-morning-voided (FMV) urine samples before clinical signs of puberty and 1–2 years earlier than the increase in serum LH (S-LH) concentrations [3]. Therefore, a longitudinal study would help to investigate the critical level of increased gonadotropin immunoreactivity as an indication of imminent manifestation of the first clinical signs of puberty and the time span for the appearance of these physical signs after such levels of gonadotropin immunoreactivity are first observed.

In this regard, we hypothesized that investigating gonadotropin immunoreactivity originating from nocturnal GnRH pulses in FMV urine samples and comparing them with LNV urine levels (baseline) may provide clues to possible cut-off values between early prepuberty and late prepuberty as well as between late prepuberty and early puberty. Because of its practicality and non-invasiveness, FMV urine is an excellent tool to monitor the activity of nocturnal pulsatility in late prepubertal children whose onset of puberty is imminent. Moreover, such an exploratory study may provide valuable information about the physiology of prepubertal changes that may indicate imminent puberty and an opportunity to assess pubertal development already before physical signs emerge.

In this exploratory study, we aimed to investigate the nocturnal changes in the immunoreactivity levels of urinary gonadotropins between early and late prepubertal stages as well as between those of puberty in FMV and LNV urine samples from 30 girls, of whom those who were prepubertal were further studied through follow-up visits within the 1-year period from the start of the study.

## Materials and methods

### Subjects

Thirty girls (aged between 8 and 15 years) attending the outpatient clinics were recruited as volunteers to provide urine samples just before bedtime (last-night-voided; LNV) and immediately after waking up (first-morning-voided; FMV). All patients were prospectively enrolled in the study according to the following inclusion criteria: (1) no established diagnosis or signs or symptoms of an underlying endocrinologic, metabolic, oncologic, or nephrologic disorder, (2) no chronic disease (severe malnutrition, congenital heart disease, chronic respiratory disease, diabetes, chronic kidney disease, muscle and nervous system disease, or metabolic disease) or immunodeficiency, and (3) no recent history of medications or hormonal contraceptives that could have affected renal function or the HPG axis.

To ensure compliance with the inclusion criteria, all subjects underwent routine urinalysis, complete blood count, ALT, AST, total bilirubin, direct bilirubin, BUN, creatinine, and thyroid function tests. In addition, serum estradiol was determined in serum. Physical examination of each subject was performed after taking a thorough history, particularly regarding growth and puberty. Name, sex, height, weight, date of birth, and age at menarche in girls as well as any reported previous diagnoses or treatments if any were recorded for each subject at the time of recruitment before reaching a decision to include or exclude them. Pubertal development was based on the Tanner stage [9]. Patients were enrolled to allow the inclusion of approximately 10 patients in each of the following categories: prepubertal, early pubertal (premenarcheal; EP), and late pubertal (postmenarcheal; (LP). The above-mentioned categories were filled with 10, 8, and 12 volunteers, respectively, at the end of the recruitment process. The prepubertal subjects were examined every 4–6 months for the next year, at the end of which five of them were classified as early prepubertal (EPP) and five others as late prepubertal (LPP) as the latter group showed clinical signs of puberty by the end of the 1-year follow-up period.

### Study design

The study was conducted at the Children’s Hospital, University of Helsinki, Finland, and the Department of Pediatrics, Dokuz Eylül University Hospital, İzmir, Türkiye. The study protocol was approved by the respective ethics committees of both university hospitals. Laboratory examinations of the samples were performed at the Department of Clinical Chemistry, University of Helsinki, Finland. Each subject provided two urine samples for gonadotropin measurements, one just before bedtime (LNV urine sample) and the other immediately upon awakening the next morning (FMV urine sample). The LNV and FMV urine samples were collected in 100-mL jars and stored separately at + 4 °C before being brought to the hospital. An aliquot was used for gonadotropin analysis. Urine samples were stored at + 4 °C for up to 10 days prior to analysis.

### Assays

The immunofluorometric assays (IFMA) employed in this study are commercially available sandwich assays using monoclonal antibodies (AutoDELFIA hFSH and hLH (the latter formerly known as LHspec), Wallac, PerkinElmer Finland Oy). One antibody is immobilized on a microplate well, and the other is labeled with a europium chelate. Both the capture and the detection antibody are directed against the ß-subunit of LH and recognize different, distinct epitopes [10]. This LH assay, specifically designed to detect intact LH and LHβ, but not human chorionic gonadotropin, also measures LHβcf, as shown in our previous study [5]. Therefore, the hLH assay in this study measured total urinary LH-ir derived from the intact LH, LHβ, and LHβcf. Assays were performed according to the manufacturer’s instructions. A sample volume of 25 μL was used for urine assays. The total assay volume was 225 μL. The assays were calibrated against the WHO Second International Standard for Pituitary LH for Immunoassay (80/552) and the Second International Reference Preparation of Pituitary FSH/LH (78/549), respectively. The limits of detection calculated using the measured limits of both the blank and the replicate of a sample known to contain a low concentration of the analyte for the urinary LH (U-LH) and urinary FSH (U-FSH) assays were 0.015 IU/L and 0.018 IU/L, respectively [11]. The intra-assay and inter-assay mean CVs for the U-LH and U-FSH assays were 5.2 and 6.4% and 2.3 and 5.7%, respectively [3]. The intra-assay coefficients of variation for both assays were < 2% at levels between 3 and 250 IU/L and approximately 10% at 0.3 IU/L. The inter-assay coefficient of variation was < 3% at 4–18 IU/L for both assays [4]. Hormone concentrations were not corrected for variations in urinary excretion rate (such as urine density or creatinine) because the correlation with serum levels has not been improved and may even be impaired by overcorrection in very dilute urine samples [1]. Serum samples were analyzed for estradiol by RIA assays (TKE2-7 Coat-A-Count kits from Diagnostic Products Corporation, LA, USA). The detection limit for the estradiol assay was 20 pmol/L. The intra-assay and inter-assay coefficients of variation were 4.0 and 4.9% for the estradiol assay.

### Statistics

This study was designed as an exploratory study to generate new hypotheses, and therefore, no formal power calculations were performed. Differences were considered statistically significant when *P* < 0.05. Overnight differences in urinary gonadotropins or their respective ratios were calculated as the FMV-to-LNV ratio, depicting the overnight fold change in the parameter of interest. The Kruskal-Wallis test was chosen to determine any differences between early prepubertal, late prepubertal, early pubertal, and late pubertal groups or combinations of these groups. The Mann-Whitney *U* test was used to analyze the significance of the difference between two given independent groups at a time testing whether they represent the same or a similar population, while the independent-samples median test was used to specifically analyze the significance of the difference between the medians of these groups. Receiver operating characteristic (ROC) analysis was used as the statistical tool to calculate different cut-off points for predicting the onset of puberty within 1 year with different levels of sensitivity and specificity. The area under the ROC curve (AUC) was used as a summary measure that essentially averaged the diagnostic accuracy across the range of test values. An AUC equaling 0.5 was considered the ROC curve equivalent to random chance, and 1.0 was considered perfect accuracy. An estimated AUC of less than 0.5 indicates a probability worse than chance.

## Results

### Changes in urinary gonadotropin concentrations during pubertal development

**Fig. 1:**
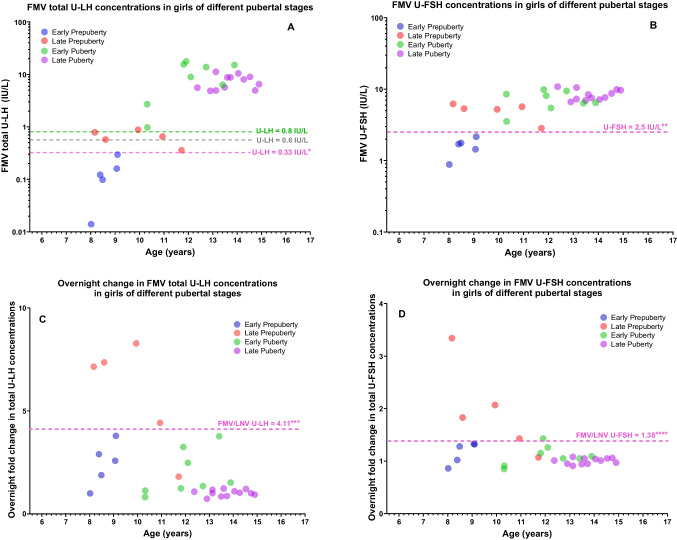
**A**, **B** Age-related course of first-morning-voided (FMV) urinary total luteinizing hormone (U-LH) and urinary follicle-stimulating hormone (U-FSH) concentrations in girls at different prepubertal and pubertal stages. **C**, **D** Age-related course of the change from last-night-voided (LNV) to FMV total U-LH and U-FSH concentrations in girls at different prepubertal and pubertal stages. The results of the ROC curve analysis corresponding to the asterisk-marked cut-off values in **A**–**D** are as follows: *AUC: 1.00, sensitivity 100.0%, and specificity 100.0%; **AUC: 1.00, sensitivity 100.0%, and specificity 100.0%; ***AUC: 0.84, sensitivity 80.0%, and specificity 100.0%; ****AUC: 0.88, sensitivity 80.0%, and specificity 100.0%

**Fig. 2:**
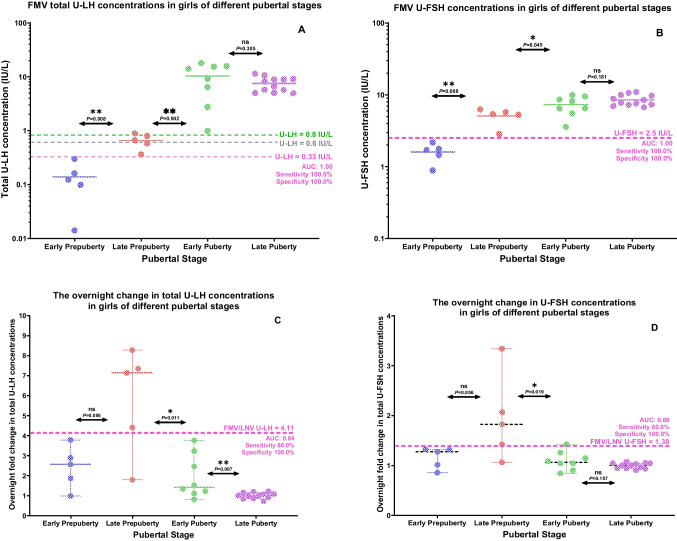
**A**, **B** The difference in first-morning-voided (FMV) urinary total luteinizing hormone (U-LH) and urinary follicle-stimulating hormone (U-FSH) concentrations between adjacent groups of girls at different prepubertal or pubertal stages. **C**, **D** The change in total U-LH and U-FSH concentrations during the course of overnight sleep between adjacent groups of girls of different prepubertal or pubertal stages

FMV total U-LH concentrations were below 0.33 IU/L in all five EPP girls and at or above the 0.6 IU/L level in four out of the five LPP girls (Fig. 1A). Interestingly, two of these four LPP girls with a FMV total U-LH concentration at or above the 0.6 IU/L were 8.2 and 8.6 years of age, i.e., in the age range of the EPP girls in this study (Fig. 1A). In addition, the FMV U-FSH concentrations were below 2.5 IU/L in all five EPP girls and above this level in all five LPP girls (Fig. 1B). The total U-LH-ir and U-FSH-ir levels were significantly higher in LPP than in EPP and similarly higher in EP than in LPP girls; however, the above-mentioned urinary gonadotropin immunoreactivity levels were not higher in LP than in EP girls (Fig. 2A, B).

### Changes in urinary gonadotropins during overnight sleep in girls of different pubertal stages

**Fig. 3:**
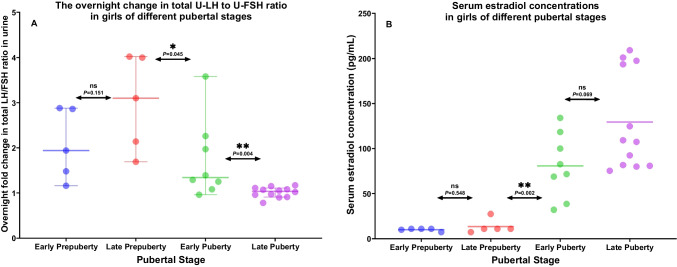
**A** The overnight change in total luteinizing hormone (U-LH) to follicle-stimulating hormone (U-FSH) immunoreactivity ratio between adjacent groups of girls of different prepubertal or pubertal stages. **B** The difference in serum estradiol concentrations between adjacent groups of girls of different prepubertal or pubertal stages

Overnight increases in total U-LH-ir and U-FSH-ir levels were significantly higher in pubertal girls than those in prepubertal girls (*P* < 0.001 and *P* = 0.005, respectively). The increase in both total U-LH and U-FSH immunoreactivity (total U-LH-ir or U-FSH-ir) levels during the course of overnight sleep in LPP girls was higher than that in EPP girls, but not at significant levels (*P* = 0.095 and *P* = 0.056), with only five subjects in each subgroup (Fig. 2C, D). Compared to the late prepubertal subjects, lower levels of overnight change in total U-LH-ir and U-FSH-ir were observed in early pubertal girls (*P* = 0.011 and *P* = 0.019, respectively). This decreasing trend in overnight change continued in total U-LH-ir levels further toward late puberty (*P* = 0.007) (Fig. 2C). Similarly, the overnight change in the total U-LH-ir-to-U-FSH-ir ratio was lower in EP than in LPP girls and likewise lower in LP than in EP girls (*P* = 0.045 and *P* = 0.004, respectively) (Fig. 3A). The overnight increase in total U-LH-ir-to-U-FSH ratio during sleep was not significantly higher in LPP girls than that in EPP girls, but this increase during overnight sleep was less than 3-fold in all the five early prepubertal girls and more than 1.7-fold in all late prepubertal girls (Fig. 3A).

### Changes in serum estradiol concentrations during pubertal development

The increase in the concentrations of all these steroid hormones was significant only across subgroups of LPP to EP girls, but not across other groups or subgroups (prepubertal vs. pubertal, EPP vs. LP, and EP vs. LP) (Fig. 3B).

### Predictability of onset of puberty within 1 year among prepubertal girls based on the longitudinal data

ROC analysis revealed that the FMV total U-LH and FMV U-FSH concentrations at or above 0.33 IU/L and 2.50 IU/L, respectively, were excellent predictors of the onset of puberty within 1 year (100% sensitivity, 100% specificity, AUC: 1.00, and *n* = 10, for both) (Table 1 and Figs. 1A, B and 2A, B). FMV/LNV total U-LH and FMV/LNV U-FSH ratios 4.11 and 1.38, respectively, were also good predictors of the onset of puberty within 1 year (80% sensitivity, AUC: 0.84 and 100% specificity, AUC: 0.88, respectively; *n* = 10 for both) (Table 1 and Figs. 1C, D and 2C, D). Other parameters of interest in this study were far from being predictors of the onset of puberty with rather low levels of either sensitivity or specificity or both (Table 1).

**Table 1:**
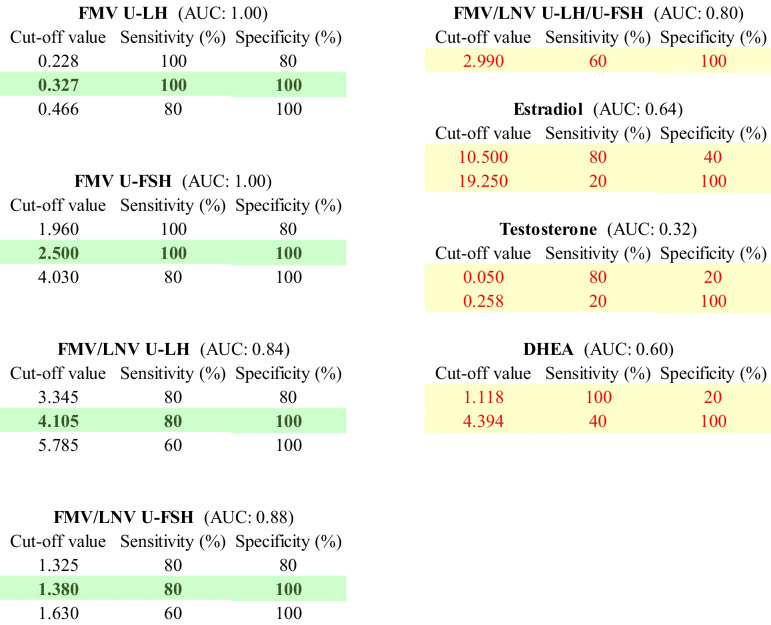
ROC analysis results for the prediction of onset of central puberty within 1 year by different parameters and their respective levels to reflect the cut-off values that perform best in terms of sensitivity and specificity. The most optimal cut-off values for qualified and insufficient parameters are highlighted in green and yellow, respectively. AUC values are provided in parentheses

### Predictability of onset of puberty based on cross-sectional data

ROC analysis revealed that the FMV total U-LH concentration at or above 0.83 IU/L was a good predictor of puberty when comparing prepubertal and pubertal girls (100% sensitivity, 90% specificity, AUC: 1.00, and *n* = 10 and *n* = 20, respectively) (Figs. 1A and 2A). ROC analysis showed that FMV total U-LH concentration at or above 0.93 IU/L was a good predictor of the onset of puberty when comparing LPP and EP girls (100% sensitivity, 100% specificity, AUC: 1.00, and *n* = 5 and *n* = 8, respectively).

### Predictability of imminent menarche based on cross-sectional data

ROC analysis revealed that overnight fold change in U-LH-ir levels and in U-LH/U-FSH ratio at or below 1.24 and 1.21, respectively, was a specific predictor of menarche when comparing EP and LP girls (75% sensitivity for both; 91.7 and 100% specificity, respectively; AUC: 0.85 and 0.88, respectively, *n* = 8 and *n* = 12, respectively, for both) (Figs. 2C and 3A).

### Overall changes in urinary gonadotropin and serum estradiol concentrations during pubertal development

Table 2 presents statistical information on FMV and LNV urinary gonadotropin concentrations and their ratios along with the concentrations of estradiol in girls at different stages of pubertal development (EPP, LPP, EP, and LP).

**Table 2:** First-morning-voided (FMV) and last-night-voided (LNV) urinary luteinizing hormone (LH) and follicle-stimulating hormone (FSH) concentrations (IU/L) and their ratios along with concentrations of estradiol in early prepubertal (EPP), late prepubertal (LPP), early pubertal (EP), and late pubertal (LP) girls

		Number	Min.	Max.	Mean	Std. Error	Std. Deviation	Variance
EPP	FMV U-LH	5	0.01	0.30	0.14	0.05	0.10	0.01
	FMV U-FSH	5	0.88	2.16	1.59	0.21	0.47	0.22
	LNV U-LH	5	0.01	0.08	0.05	0.01	0.02	0.00
	LNV U-FSH	5	1.02	1.67	1.36	0.14	0.30	0.09
	FMV/LNV U-LH	5	1.00	3.79	2.43	0.47	1.06	1.11
	FMV/LNV U-FSH	5	0.86	1.33	1.16	0.09	0.21	0.04
	FMV/LNV U-LH/U-FSH	5	1.16	2.88	2.06	0.35	0.79	0.62
	Estradiol	5	7.34	11.01	10.08	0.71	1.59	2.53
LPP	FMV U-LH	5	0.36	0.88	0.65	0.09	0.20	0.04
	FMV U-FSH	5	2.84	6.22	5.06	0.58	1.30	1.69
	LNV U-LH	5	0.08	0.20	0.13	0.02	0.05	0.00
	LNV U-FSH	5	1.86	3.98	2.79	0.35	0.77	0.60
	FMV/LNV U-LH	5	1.81	8.28	5.80	1.19	2.66	7.06
	FMV/LNV U-FSH	5	1.07	3.34	1.95	0.39	0.87	0.76
	FMV/LNV U-LH/U-FSH	5	1.69	4.02	2.99	0.47	1.06	1.13
	Estradiol	5	7.34	27.53	13.58	3.56	7.96	63.34
EP	FMV U-LH	8	0.98	17.72	10.16	2.24	6.33	40.09
	FMV U-FSH	8	3.54	9.88	7.23	0.76	2.15	4.63
	LNV U-LH	8	0.86	12.58	5.96	1.55	4.39	19.29
	LNV U-FSH	8	3.90	9.98	6.68	0.79	2.24	5.02
	FMV/LNV U-LH	8	0.82	3.77	1.94	0.38	1.09	1.18
	FMV/LNV U-FSH	8	0.85	1.43	1.10	0.07	0.19	0.04
	FMV/LNV U-LH/U-FSH	8	0.96	3.58	1.72	0.31	0.87	0.76
	Estradiol	8	32.12	133.99	80.76	12.68	35.85	1285.57
LP	FMV U-LH	12	4.86	11.22	7.41	0.65	2.26	5.13
	FMV U-FSH	12	6.64	10.86	8.45	0.43	1.47	2.17
	LNV U-LH	12	4.90	10.12	7.24	0.52	1.80	3.22
	LNV U-FSH	12	6.86	10.76	8.41	0.36	1.26	1.60
	FMV/LNV U-LH	12	0.74	1.23	1.02	0.04	0.15	0.02
	FMV/LNV U-FSH	12	0.91	1.08	1.00	0.02	0.06	0.00
	FMV/LNV U-LH/U-FSH	12	0.78	1.17	1.01	0.03	0.11	0.01
	Estradiol	12	75.26	209.25	129.42	15.71	54.43	2962.5

## Discussion

This is the first report, to the best of our knowledge, on the performance of urinary gonadotropins in a cohort of children that have been on follow-up for signs of puberty progression. Longitudinal cohort studies in peripubertal children are scarce, and there is a significant knowledge gap in this area. The results of this study confirm that FMV U-LH determinations, which reflect the integrated LH pulse secretion into the bloodstream during sleep, are a sensitive method to detect HPG axis activation already before the manifestation of clinical signs of puberty. This has been demonstrated in previous cross-sectional and longitudinal studies on the clinical aspects of urinary gonadotropin measurements [3, 4, 12–14]. The widespread use of non-invasive and sensitive methods for the assessment of gonadotropin secretion is becoming increasingly important because of the continuing trend toward an earlier onset of puberty in both sexes, particularly in girls, mainly due to environmental factors; indeed, the proportion of girls with non-progressive isolated thelarche has increased during recent decades among early maturing girls [15, 16]. Evaluation of gonadotropin secretion activity by quantification of FMV total U-LH concentrations by sensitive assays can be beneficial for differentiation between isolated thelarche and progressive central precocious puberty.

In addition, abnormal timing of pubertal development has been reported to be associated with adverse health and psychosocial outcomes. For example, early age at menarche is associated with an increased risk of obesity, type 2 diabetes [17], and cardiovascular disease [18] later in life. Other reported associations with early menarche include increased risk of breast cancer [19] and all-cause mortality [20]. These are not only important for the individual, but also have potentially large public health implications, especially given the secular trend toward earlier onset of puberty [21, 22].

In this study, it was possible to differentiate between the EPP and LPP girls by both FMV U-LH and U-FSH determinations before the LPP girls showed physical signs of puberty. FMV total U-LH concentrations at or above 0.8 IU/L were shown to be a good predictor of clinical signs of puberty across prepubertal and pubertal girls according to the results of this study. Our study results also showed that FMV total U-LH concentrations above 0.3 IU/L were an excellent predictor of imminent HPG axis activation because there is naturally a time lag between HPG axis activation and the manifestation of the physical signs of puberty. Another interesting finding was that the FMV total U-LH concentrations were not only above the aforementioned cut-off value of 0.3 IU/L in all the five LPP girls differentiating them from all the five EPP girls, but also at or above 0.6 IU/L in four of the five LPP girls, two of whom were even younger than some of the EPP girls. These findings imply that the cut-off values for FMV total U-LH concentrations signifying forthcoming HPG axis activation and the manifestation of clinical signs of puberty are approximately 0.3 and 0.8 IU/L; accordingly, the FMV total U-LH concentration to indicate HPG axis activation, i.e., the onset of central puberty, should be in the 0.3–0.8 IU/L range, presumably around 0.6 IU/L according to the results of this limited semi-longitudinal study. Thus, ROC curve analyses from this exploratory study need to be replicated in larger cohorts.

A novel aspect of this study design is also the use of the overnight fold change (FMV/LNV ratio) in U-LH-ir and U-FSH-ir levels as a conceptual analogy to a GnRH stimulation test setting. LNV and FMV urine gonadotropin concentrations represented the basal level and the AUC of the GnRH test, respectively. The overnight fold change in total urinary LH or FSH concentrations, which reflects the change in the respective gonadotropin pulse activity during the whole course of overnight sleep, appears to be one of the parameters that add value to the assessment of pubertal development. In this particular study with a rather limited number of EPP and LPP subjects, detection of a transient increase in the overnight fold change in total U-LH-ir levels did not appear to be superior to FMV U-LH determinations alone in predicting imminent puberty. However, based on our results, since elevated overnight change in U-LH appears to be a parameter that is uniquely associated with LPP, but not with EPP, EP, or LP girls, it can be used as an adjunct parameter to evaluate the central activation of puberty in cases that remain borderline according to FMV total U-LH results. While our results suggest that the value of overnight change in U-LH is of limited value in the clinical setting, future studies should clarify whether the change can add sensitivity and specificity to FMV U-LH in different clinical scenarios. This phenomenon reflects the well-known physiological pattern of increased gonadotropin secretion exclusively during the night in early puberty, followed by loss of diurnal variation in the later stages of puberty. In addition, the overnight fold change in total U-LH-ir levels or the total U-LH/U-FSH ratio appears to be predictive of imminent menarche, as the increase in total U-LH concentration during the course of overnight sleep until morning is significantly higher in EP subjects when compared to that in LP subjects (Fig. 2C). Future studies may determine whether the transition from the premenarcheal stage to menarche is associated with a specific pattern of urinary gonadotropin secretion.

Furthermore, little is known about the critical ratio between LH and FSH during the pubertal transition in both sexes despite a large number of previous studies since the 1970s on the relevance of the LH:FSH ratio in the physiology as well as in the diagnostics of pubertal disorders [23–25]. An earlier study of ours revealed the sex difference in U-LH/U-FSH ratio in peripubertal boys and girls as well as at different pubertal stages, confirming the relative FSH dominance in girls compared to boys [4]. The results of the current study suggest that imminent menarche may be related to an overnight increase rate in total U-LH/U-FSH ratio or in FMV total U-LH-ir levels remaining at or below 21 and 24%, respectively, which should be confirmed by further longitudinal research to reveal the estimated time to menarche at the same time.

The main strength of the current study is its semi-longitudinal design which enabled clinical signs of puberty to be detected in some initially prepubertal children over the course of a 1-year follow-up. The high sensitivity and specificity of the test method were another important strength of the current study. On the other hand, the small sample size and the lack of serum gonadotropin measurements were major limitations of this study. In addition, mass spectrometric determination of estradiol and testosterone would have been a better alternative to RIA, the sensitivity of which may still be too low for the determination of these steroid hormones in the pediatric age group.

It can be concluded that FMV total U-LH and U-FSH above 0.3 IU/L and 2.5 IU/L, respectively, may serve as cut-off values to predict the manifestation of the clinical signs of puberty within 1 year. Moreover, FMV total U-LH above 0.8 IU/L in our cohort was exclusively found in those exhibiting clinical signs of puberty and may serve as a threshold that differentiates girls with central precocious puberty from those still prepubertal. The suggested range and cut-off for FMV total U-LH concentrations (0.3–0.8 IU/L and 0.6 IU/L, respectively) signifying the critical FMV total U-LH-ir levels associated with the release of the central brake on the GnRH pulse generator should be verified by further larger studies involving early morning serum LH and FSH determinations and comparisons against the gold standard (such as early morning serum LH concentration cut-off at 0.3 IU/L) in children and adolescents of both sexes. Thereafter, FMV total U-LH and U-FSH determinations can be used as the first-line tool in the evaluation of pubertal development as well as for screening and diagnostics of pubertal disorders; hence, the need for invasive GnRH stimulation tests or early morning serum LH determinations may be decreased substantially. In addition, an overnight increase in U-LH-ir levels or in the U-LH/U-FSH ratio remaining at or below 1.2-fold in an early pubertal girl may be a sign of imminent menarche, the presumed timing of which can be unraveled by longitudinal studies. Our hypothesis that integrated gonadotropin activity during overnight sleep is reflected as an increase in integrated total U-LH or U-FSH immunoreactivity during overnight sleep also requires further investigation in a larger cohort with power analysis for conclusive ROC analyses.

## Data Availability

The results data generated during the current study are available from the corresponding author on reasonable request.
