# Correction: Quantification of overnight urinary gonadotropin excretion predicts imminent puberty in girls: a semi-longitudinal study

**DOI:** 10.1007/s42000-023-00512-z

**Published:** 2023-11-28

**Authors:** And Demir, Atilla Büyükgebiz, Adem Aydin, Matti Hero

**Affiliations:** 1 Pediatric Research Center, New Childrenʼs Hospital, University of Helsinki and Helsinki University Hospital, Biomedicum 2 C, 6th Floor, Tukholmankatu 8 A, FIN-00290 Helsinki, Finland; 2 Department of Pediatrics, Division of Pediatric Endocrinology, Demiroğlu Bilim University, Istanbul, Türkiye; 3 Department of Pediatrics, Dokuz Eylül University Faculty of Medicine, İzmir, Türkiye

**Correction: Hormones**

10.1007/s42000-023-00499-7

In this article, at panel C in Figure 2, the word “U-FSH” in the title of the graph should be changed to “total U-LH”.

In Results section, under the title “Predictability of onset of puberty based on cross sectional data”, the text “(Figs. 1A and 2A)” should be added to the end of the first sentence.

In Results section, under the title “Predictability of imminent menarche based on cross sectional data”, the text “(Figs. 2C and 3A)” should be added to the end of the first sentence.

The original article has been corrected.

